# Ischemic stroke in Morocco: a systematic review

**DOI:** 10.1186/s12883-019-1558-1

**Published:** 2019-12-30

**Authors:** Ahmed Kharbach, Majdouline Obtel, Laila Lahlou, Jehanne Aasfara, Nour Mekaoui, Rachid Razine

**Affiliations:** 1 Laboratory of Social Medicine (Public Health, Hygiene and Preventive Medicine), Faculty of Medicine and Pharmacy of Rabat, Mohamed V University, 10100 Rabat, Morocco; 2 Laboratory of Biostatistics, Clinical Research and Epidemiology (LBRCE), Faculty of Medicine and Pharmacy of Rabat, Mohamed V University, 10100 Rabat, Morocco; 3 Department of Neurology, International Cheikh Khalifa University Hospital, Mohammed VI University of Health Sciences (UM6SS), 82403 Casablanca, Morocco; 4 Faculty of Medicine and Pharmacy of Agadir, Ibn Zohr University, 80060 Agadir, Morocco; 5 Pediatric medical emergencies service of the Children Hospital, 10100 Rabat, Morocco

**Keywords:** Ischemic stroke, Trial of ORG classification 10,172 in acute stroke treatment classification, Prehospital delay, Thrombolysis, Morocco

## Abstract

**Background:**

The aim of this systematic review is to determine the epidemiological and etiological profiles, the influential factors of the prehospital delay, thrombolysis management, the acute and 3-month mortality rate and the genetic aspect of ischemic stroke in Morocco.

**Methods:**

The present work is a systematic review that was conducted according to the recommendations of the “Preferred reporting items for systematic reviews and meta-analysis”. We used Pubmed, Sciencedirect, Scopus, Clinicalkey, and Google scholar databases for the raking of the gray literature during the time frame 2009 and 2018. The protocol of the review was registered in the PROSPERO register (CRD42018115206).

These studies were analyzed based on: Age, sex ratio, risk factors, etiological profile according to Trial of ORG classification 10,172 in Acute Stroke Treatment, prehospital delay average and its influential factors, thrombolyzed patients’ proportion, acute and 3-month mortality and the genetic factors of ischemic stroke in Morocco.

**Results:**

Twenty-nine (*n* = 29) studies were selected. The average age ranged from 49 ± 15.2 to 67.3 ± 9.9 years old. Moreover, we reported male predominance within all ages in 13 studies. High blood pressure, diabetes, smoking and heart disease were the four identified main risk factors by the prementioned studies. Atherosclerosis and cardioembolic were the main described etiologies of cerebral ischemia, and the average prehospital time ranged from 26 to 61.9 h. The proportion of thrombolysed patients ranged from 1.8% to 2.9%, the mortality rate varied in the acute phase from 3 to 13%, and the 3-month mortality ranged from 4.3 to 32.5%. It is also important to highlight that most of these studies, which were conducted in hospital environment, have a reduced sample size and no confidence interval.

**Conclusions:**

Ischemic stroke is affecting more likely the young population with male predominance. Moreover, the long prehospital delay and the low proportion of thrombolysed patients are alarming. This indicates the need to investigate in depth the key factors influencing the access to care for Moroccan patients in order to improve the management of this neurologic deficit in Morocco.

## Background

Stroke has become a major public health concern and a real growing burden on African countries [1, 2], regarding its cost on the social, psychological, and economic levels [3]. The incidence of stroke continues to increase in developing countries, including the North African region [4].

From an etiological point of view, previous studies have described multiple causes of stroke [5]. According to the epidemiological survey conducted in the two metropolitan Moroccan cities (Casablanca and Rabat), ischemic stroke (IS) represented 70.9% of all types of stroke [6]. Multidisciplinary and fast approaches are highly required in term of therapeutic management since they are key determinants of the prognosis and evolution of the disease [7]. In addition, a major evolution in IS care management is occurring during the last years. The two main improvements are the approval of intravenous thrombolysis and the intra-arterial mechanical thrombectomy [8]. Stroke is thus considered as a critical challenge in terms of prevention and patients’ management in acute phase in order to improve the mortality and morbidity of this pathology [7, 9].

In the context of low-and-middle income countries, and despite the enormous burden of stroke, only 15% of medical and fundamental research is dedicated to study and explore this medical condition compared to 85% in high-income countries [10]. Furthermore, in Morocco, there is a lack of data concerning the temporal patterns of the incidence or the long-term evolution of this debilitating disease [11].

Since ischemic stroke is largely preventable, it is important to study the epidemiological and other aspects of care to reduce the incidence rate and the resulting burden on our kingdom. It is important to highlight that this is the first systematic review of the literature on stroke to be conducted in Morocco. In this respect, the objective of this work was to evaluate, through the systematic review, the epidemiological and etiological profiles, influencing factors of the prehospital delay, thrombolysis management, mortality rate in the acute phase and at 3 months, along with the genetic aspect of IS in Morocco.

## Methods

### Research strategy

This systematic review was conducted in accordance with the methodological criteria of the Preferred reporting items for systematic reviews and meta-analyses (PRISMA) [12]. The protocol has been previously registered and published (PROSPERO: CRD42018115206/http://www.crd.york.ac.uk/PROSPERO/display_record.php?ID=CRD42018115206).

This is a systematic review of the literature by adopting a multisource research strategy, consulting the databases of Pubmed, Sciencedirect, Scopus, Clinicalkey, and Google scholar for raking gray literature (National Scientific Research Works such as Master theses and PhD dissertations of medicine) between 2009 and 2018 (Last questioning on November, the 29th 2018).

The key words used were as follows: (Ischemic stroke) or (cerebral infarction) or (Cerebral ischemia) or (ischemic stroke) or (ischemic attack) or (Cerebral ischemia) or (Cerebral infarction) and (Morocco) or indicating specific cities of the kingdom.

Two authors (KA and OM) independently verified titles and abstracts to identify the to-be included studies. Complete articles of the potential studies were downloaded for a more detailed evaluation and the list of references in all relevant articles were examined for additional documents as well as for the citing papers. No restrictions were made on the language of publication.

### Inclusion and exclusion criteria

1. Studies were conducted in Morocco and published after 2009 concerning IS.
2. Studies on the following data: age, sex ratio, risk factors, etiologic profile, prehospital delay and the management of cerebral ischemia including thrombolysis.
3. Studies on genetic polymorphisms related to the occurrence of cerebral ischemia in Morocco.
4. Studies on IS in patients aged less than 15 years old where cerebral venous thromboses have been excluded.

### Extraction and analysis of data

The data extracted from the identified documents were the following: average age, sex ratio, risk factors, etiological profile according to the Trial of ORG classification 10,172 in Acute Stroke Treatment (TOAST) classification, prehospital delay, stroke management (thrombolysis), mortality rate at the acute phase and at 3 months according to the modified Rankin score, and genes involved in the occurrence of cerebral ischemia in Morocco.

### Risk of bias in individual studies

Bias risk assessment was conducted by two authors independently through the use of “Quality Assessment Tool for Case Series Studies”, “Quality Assessment Tool for Cross-Sectional Studies” and “Newcastle-Ottawa Quality Assessment Scale Case-Control Studies”.

Two reviewers (KA and OM) assessed the methodological quality of the studies independently and then the agreement between the two examiners’ results was analyzed by the Kappa statistical coefficient (κ).

## Results

**Fig. 1:**
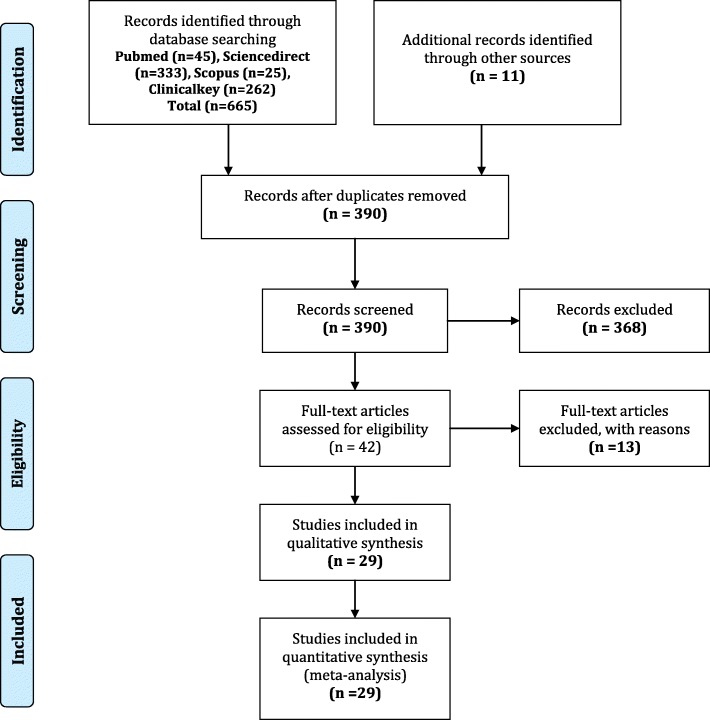
PRISMA 2009 flow diagram

Twenty-nine (*n* = 29) studies were selected (The flow chart is shown in Fig. 1). Based on study type: Full original papers (*n* = 13), conference abstracts (*n* = 6), medical theses (*n* = 10). By locations: Rabat (*n* = 5), Casablanca (*n* = 7), Casablanca and Rabat (*n* = 1), Marrakech (*n* = 7), Fez (*n* = 8), and Meknes (*n* = 1). According to the study design: (*n* = 15) were retrospective case series, (*n* = 8) were prospective case series, (*n* = 1) were prospective cross sectional study, and (*n* = 5) were prospective case-control studies. According to the age of the target population of the selected studies, (*n* = 25) studies targeted all ages combined (More than 15 years old) and (*n* = 4) studies focused on the young population (15–45 years old). All studies were hospital series.

The Kappa statistical coefficient was (κ = 0.64). The Quality Rating of “case series”, “cas-control” and “cross-sectional” studies were considered “good” with an average score of 7.5/9 (the results of the methodological evaluation of case series studies are shown in Table 1).

**Table 1:** Quality assessment tool for case series studies

Criteria	[13]	[14]	[15]	[16]	[17]	[18]	[19]	[20]	[21]	[22]	[23]	[24]	[25]	[26]	[27]	[28]	[29]	[30]	[31]	[32]	[33]	[34]	[35]
1. Was the study question or objective clearly stated?	1	1	1	1	1	1	1	1	1	1	1	1	1	1	1	1	1	1	1	1	1	1	1
2. Was the study population clearly and fully described, including a case definition?	1	1	1	1	1	1	1	1	1	1	1	1	1	1	1	1	1	1	1	1	1	1	1
3. Were the cases consecutive?	1	1	1	1	1	1	1	1	1	1	1	1	1	1	1	1	1	1	1	1	1	1	1
4. Were the subjects comparable?	1	1	1	1	1	1	1	1	1	1	1	1	1	1	1	1	1	1	1	1	1	1	1
5. Was the intervention clearly described?	1	1	1	1	1	1	1	0	0	1	1	0	1	1	1	1	1	0	1	1	1	1	1
6. Were the outcome measures clearly defined, valid, reliable, and implemented consistently across all study participants?	1	0	0	1	1	1	0	0	0	1	0	1	1	1	1	1	1	1	1	1	1	1	1
7. Was the length of follow-up adequate?	1	1	0	1	0	1	1	1	1	1	1	1	0	1	1	0	1	1	1	1	1	1	1
8. Were the statistical methods well-described?	1	0	0	0	0	1	0	0	0	0	0	0	0	0	0	0	1	0	0	1	1	0	0
9. Were the results well-described?	1	1	1	1	1	1	1	1	1	1	1	0	1	1	1	1	1	1	1	1	1	1	1
Score	9	7	6	8	6	9	7	6	6	8	7	6	7	8	8	7	9	7	8	9	9	8	8

### Mean age and sex ratio of patients with ischemic stroke in Morocco

Twenty-three studies focusing on IS in all age groups revealed an average age ranging from 49 ± 15.2 to 67.3 ± 9.9 years old. Thus, the average age is listed in the fifth and sixth decade [13, 15–18, 20, 21, 23–28, 32–41]. However, two studies did not specify the average age of patients. The first study was conducted by Chraa (2010), who reported that the age was below 45 years old in 36% of cases and more than 45 years old in 64% of cases, while the second by Bourazza et al. (2013) indicated an age between the two extremes of 24 and 104 years old [19, 30].

As for the sex ratio (*n* = 13), studies revealed a male predominance with a ratio ranging between 1.23 and 3.45 [16–19, 24–26, 36–40, 42]. A ratio of 1 has been reported only in (*n* = 3) studies [23, 40, 41]. Similarly, a slight female predominance with a ratio between 0.7 and 0.9 was reported in (*n* = 9) studies [13, 20, 21, 25, 28, 30, 32–34].

Concerning IS in early adulthood, four studies (*n* = 4) included patients aged between 15 and 45 years old. The first was a study by Mbagui (2009) with an age ranging between 15 and 45 years old, the second of Ibouajbane (2014) between 16 and 45, the third of Chraa et al. (2014) with an age between 18 and 45 and the fourth of Allaoui et al. (2018), which included all patients admitted to internal medicine under the age of 45 [14, 22, 29, 31, 43].

According to the studies of IS in young people, the average age ranged from 28.3 ± 4.2 to 39 years old (Extremes: 16–45 years old) [14, 22, 29, 31, 43].

Concerning the sex-ratio of IS in young cases, (*n* = 2) studies reported values less than 1. The first is attributable to the one by Ibouajbane (2014) with a clear female predominance (sex -ratio of 0.4) [31], and the second was performed by Allaoui et al. (2018) showing a slight female predominance with a sex ratio of 0.7 [43].

Male predominance was described by Chraa et al. (2014) with a sex ratio of 1.4 [22]. In addition, the Mbagui (2009) study did not show any significant difference between the two genders [29].

### Risk factors for ischemic stroke in Morocco according to the selected studies

Studies included in the present critical review of the literature have revealed several risk factors associated with cerebral ischemia in the local populations of interest. Indeed, high blood pressure (HBP), diabetes, smoking and heart disease were the four main risk factors listed and are as follows: HBP was reported in (*n* = 20) studies (31 to 65.4%) [13, 15, 17, 18, 20, 21, 23, 25–28, 30, 33, 35–38, 40–42], diabetes in (*n* = 20) studies (12–41.8%) [13, 15, 17, 18, 20, 21, 23, 25–28, 30, 33, 35–38, 40–42], cardiac diseases in 14 studies (7–44.3%) [13, 15, 17, 20, 21, 23, 25–28, 30, 33, 35, 42], atrial fibrillation as associated heart disease was specified in 9 studies (2,5–22%), and smoking in (*n* = 19) studies (4–41.8%) [13, 15, 17, 18, 20, 21, 23, 25–28, 30, 33, 35, 37, 38, 40–42].

In addition to these risk factors, other risk factors have been reported such as dyslipidemia in (*n* = 16) studies (0–61.8%) [13, 15, 17, 18, 20, 23, 25–28, 30, 33, 35–37, 42], obesity in (*n* = 6) studies (10.7–26.1%) [13, 17, 18, 26, 30, 35], the notion of a previous stroke was noted in (*n* = 10) hospital series (5–26.6%) [13, 15, 23, 25–28, 30, 32, 35], alcoholism in (*n* = 10) studies [13, 15, 20, 25, 32, 33, 35, 38, 40, 41], oral contraception in (*n* = 3) studies (6.6–12.2%) [30, 32, 33], and migraine in a single study at 6.5% [30].

Concerning the young population, the reported risk factors were smoking in (*n* = 4) studies (5–40.6%) [14, 22, 29, 31], HBP in (*n* = 4) studies (8% - 49.2%) [14, 22, 29, 31, 43], oral contraception in (*n* = 4) studies (12–31.2%) [14, 22, 29, 31, 43], cardiac diseases only by Chraa et al. (2014) with a percentage of 17.9% [22], diabetes in (*n* = 4) studies (7.5–13.2%) [14, 22, 29, 31, 43], migraine in (*n* = 4) studies (1.5–24%) [14, 22, 29, 31, 43], dyslipidemia in (*n* = 3) studies (0–15.3%) [22, 29, 31], alcoholism in (*n* = 3) studies (5–8%) [22, 29, 31], obesity only (*n* = 1) by the study of Ibouajbane (2014) with a percentage of 2.5% [31], previous strokes history in (*n* = 2) studies by Chraa et al. (2014) and by Ibouajbane (2014) with a percentage of 2.3% and 2.5%, respectively [22, 31], the first-degree family history of stroke was reported in a single study by Allaoui et al. (2018) with a percentage of 25% [43] and pregnancy was reported as key risk factor in (*n* = 2) studies, conducted by Mbagui (2009) and Chraa et al. (2014) with percentages of 0.9% and 1.5% respectively [22, 29] (The results are detailed in Tables 2 and 3).

**Table 2:** Risk factors for ischemic stroke in Morocco according to selected studies (First part)

Studies	Location	Age (years)	Sample size	HBP (%)	OR 95% CI	Diabetes (%)	OR 95% CI	Dyslipidemia (%)	OR 95% CI	Cardiac diseases (%)/AF (%)	OR 95% CI
MBAGUI, R., 2009 [29]	Rabat	39	93 ^YIS^	11,5	–	9,6	–	15,4	–	–	–
Abjaw, Z et al., 2009 [13]	Marrakech	62,3	84 ^IS^	40,4	–	25	–	8,3	–	14,2	–
Rhissassi et al., 2010 [34]	Fes	65,5	342 ^IS^	–	–	–	–	–	–	–	–
N A-Y AMESSAN., 2010 [15]	Rabat	59,9	46 ^IS^	51	–	30	–	7	–	25/15	–
Chraa, M., 2010 [30]	Marrakech	–	352 ^IS^	42,9	–	15,3	–	5,7	–	13,9	–
They TP et al., 2011 [40]	Casablanca	49/46,2^NS^	91 ^ISC^/182^Cl^	50,5/9,9^**†^	9,3 4,9-17,6	17,6/8,8^*†^	2,2 1,05-4,7	–	–	–	–
AZDAD, O., 2012 [33]	Fes	66,3	1300 ^IS^	40,3	–	21,5	–	3,4	–	10,5	–
RACHDI, L., 2012 [27]	Meknes	63	40 ^ISTr^	32	–	15	–	10	–	24/17	–
Bendriss, L et al., 2012 [17]	Marrakech	60,8	110 ^IS^	65,4	–	41,8	–	10	–	19,9/9	–
Chtaou, N., 2012 [25]	Fes	60,6	50 ^IS^	48	–	18	–	14	–	−/22	–
Saraya, T., 2013 [35]	Rabat	66,7	242^IS^	56,2	–	38,4	–	13	–	18,9	–
Bourazza, A., 2013 [19]	Rabat	–	1256^IS^	–	–	–	–	–	–	–	–
They TP et al., 2013 [41]	Casablanca	49/46,2^NS^	91^ISC^/182^Cl^	50,5/9,9^**Ω^	–	17,6/8,8^* Ω^	–	–	–	–	–
Ibouajbane, M., 2014 [31]	Meknes	36	40 ^YIS^	12,5	–	7,5	–	0	–	–	–
Balar, K et al., 2014 [36]	Casablanca	57	165	50,5	–	21,3	–	11	–	–	–
Chraa, M et al., 2014 [22]	Marrakech	28,3	128 ^YIS^	49,2	–	13,2	–	7,8	–	–	–
Diakite, B et al., 2014 [39]	Casablanca	56,5	165	–	–	–	–	–	–	–	–
Allaoui, A et al., 2018 [43]	Casablanca	36	25^YIS^	8	–	8	–	–	–	–	–
RACHDI., L et al., 2015 [28]	Fes	66	439 ^IS^	43	–	29	–	0	–	−/7	–
Belkouch et al., 2015 [16]	Rabat	63	13 ^IS^	–	–	–	–	–	–	–	–
Chraa, M., 2015 [21]	Marrakech	61	665 ^IS^	42,9	–	15,3	–	5,7	–	44,3	–
Diakite, B et al., 2015 [37]	Casablanca	57	170 ^IS^	50,6	–	22,9	–	9,4	–	–	–
Benkirane, N et al.,2015 [18]	C and R^‡^	59,5	157 ^IS^	59,9	–	31,2	–	61,8	–	–	–
Yonmadji, N., 2016 [32]	Fes	64,9	1184 ^IS^	39,4	–	30	–	5,8	–	9,6/2,5	–
Daouda et al., 2018 [24]	Fes	67,3	46 ^IStr^	–	–	–	–	–	–	–	–
Diakite, B et al., 2016 [38]	Casablanca	57,1	175 ^IS^	50,3	–	20,6	–	9,4	–	–	–
Chraa M et al., 2017 [20]	Marrakech	61	442 ^IS^	42,9	–	15,3	–	–	–	44,3/13,9	–
Hadi, A et al., 2018 [26]	Marrakech	66,3	230 ^IS^	61	–	41	–	9	–	28,9/9	–
Chtaou, N et al., 2016 [23]	Fes	63	52 ^IS^	31	–	12	–	8	–	27/17	–

*AF* Atrial fibrillation, *IS* Ischemic Stroke, *ISC* Ischemic stroke cases, *Cl* Controls, *NS* Not significant, *YIS* Young Ischemic Stroke, *ISTr* Ischemic stroke trombolysed, *Ob* Obesity, *M* Migraine, *HBP* High Blood Pressure, ^‡^ Casablanca and Rabat;^*^ *P*-value< 0,05; ^**^P-value< 0,001; ^†^: *P*-values obtained using Mantel–Haenszel chi-square exact test; ^Ω^ *P* value obtained using Fisher’s exact test, *OR* Odds ratio, *CI* confidence interval

**Table 3:** Risk factors for ischemic stroke in Morocco according to selected studies (Part Two)

Studies	Location	Smoking (%)	OR 95% CI	Previous stroke/ family history of stroke (%)	Alcoholism (%)	OR 95% CI	Oral contraception (%)	Others (%)
MBAGUI, R., 2009 [29]	Rabat	33,6	–	–	7,6	–	17,3	4,8
Abjaw, Z et al., 2009 [13]	Marrakech	13,1	–	17,8	13,1	–	–	10,7 ^ob^
Rhissassi et al., 2010 [34]	Fes	–	–	–	–	–	–	–
N A-Y AMESSAN., 2010 [15]	Rabat	15	–	5	2,5	–	–	–
Chraa, M., 2010 [30]	Marrakech	25,3	–	11,9	–	–	12,2	19,88 ^ob^
They TP et al., 2011 [40]	Casablanca	22^C^/7,1^Cl*†^	3,7 1,7-7,8	–	15,4 ^C^ /2,2 ^Cl**†^	8,8 2,6-	–	–
AZDAD, O., 2012 [33]	Fes	12,9	–	–	0,2	25,4	6,64	–
RACHDI, L., 2012 [27]	Meknes	13	–	7	–	–	–	–
Bendriss, L et al., 2012 [17]	Marrakech	35,4	–	–	–	–	–	15,4^ob^
Chtaou, N., 2012 [25]	Fes	14	–	12		–		
Saraya, T., 2013 [35]	Rabat	41,8	–	4,7	4,13	–	–	19,4^ob^
Bourazza, A., 2013 [19]	Rabat	–	–	–	–	–	–	–
They TP et al., 2013 [41]	Casablanca	22^C^/7,1^Cl*Ω^	–	–	15,4^C^/2,2^Cl**Ω^	–	–	–
Ibouajbane, M., 2014 [31]	Meknes	5	–	2,5	5	–	17,5	2,5^ob^/5^M^
Balar, K et al., 2014 [36]	Casablanca	–	–	–		–		
Chraa, M et al., 2014 [22]	Marrakech	40,6	–	2,3	8	–	31,2	1,56 ^M^
Diakite, B et al., 2014 [39]	Casablanca	–	–	–	–	–	–	–
Allaoui, A et al., 2018 [43]	Casablanca	32	–	−/25	–	–	12	24^M^
RACHDI., L et al., 2015 [28]	Fes	7	–	7	–	–	–	–
Belkouch et al., 2015 [16]	Rabat	–	–	–	–	–	–	–
Chraa, M., 2015 [21]	Marrakech	25,3	–	–	5	–	–	–
Diakite, B et al., 2015 [37]	Casablanca	34,1	–	–	–	–	–	–
Benkirane, N et al.,2015 [18]	C and R^‡^	31,8	–	–	–	–	–	26,1^Ob^
Yonmadji, N., 2016 [32]	Fes	18,6	–	8,4	0	–	8,8	–
Daouda et al., 2018 [24]	Fes	–	–	–	–	–	–	–
Diakite, B et al., 2016 [38]	Casablanca	33,7	–	–	8	–	–	–
Chraa M et al., 2017 [20]	Marrakech	25,3	–	–	–	–	–	–
Hadi, A et al., 2018 [26]	Marrakech	33,3	–	9,2	–	–	–	20 ^Ob^
Chtaou, N et al., 2016 [23]	Fes	4	–	6	–	–	–	–

*C* Cases, *Cl* Controls, *IS* Ischemic Stroke, *YIS* Young Ischemic Stroke, *ISTr* Ischemic stroke trombolysed, *Ob* Obesity, *M* Migraine, *HBP* High Blood Pressure

^‡^Casablanca and Rabat; ^*^P-value< 0,05; ^**^P-value< 0,001; ^†^*P*-values obtained using Mantel–Haenszel chi-square exact test; ^Ω^*P* value obtained using Fisher’s exact test

### Genetic risk factors for ischemic stroke in Moroccan studies

The present systematic review includes only (*n* = 5) studies focusing on the genetic factors associated with IS in Morocco.

The first study by They et al. (2011) suggested that the *MTHFR C677T* variant could be a determinant of the atherothrombotic event of IS in Morocco [40]. The same team, They et al. (2013) demonstrated an interaction between *MTHFR C677TT* and *F2 20210GA* polymorphisms linked to an increased risk of IS [35]. The third study by Diakite et al. (2014) suggested another statistically significant association between *G894 T* polymorphism at the level of *eNOS* gene and IS in the recessive, dominant and additive models [39].

In addition, another genetic study by Diakite et al. (2015) evaluated the association of the *FVF C2491T* mutation with the risk of IS, suggesting that carriers of the mutated T allele were associated with a high risk of IS. But this risk was 8.95 times higher when the subject had the TT genotype (*P* <  0.0001) and 4.08 times higher with the CT genotype, and they concluded that the *FVF C2491T* mutation could be a genetic risk factor for IS in the Moroccan population [37].

The fourth genetic research was conducted by Diakite et al. (2016) on *T-1131C APOA5* polymorphism; he observed a modest risk of IS with CC and C alleles. In addition, the same study explored also the risk of IS related to *SG13S114 ALOX5AP* and showed a significant association with TT and T alleles. Despite the reduced sample size, variants of *T-1131C APOA5* and *SG13S114* could be considered as an independent genetic risk factor IS in the Moroccan population [38]. Furthermore, the fifth study by Balar (2014), showed that *MTHFR* gene (patients with *MTHFR* CT/ TT patients without CT/TT) and other factors (sex, age, HBP, diabetes, smoking, alcoholism, dyslipidemia) did not reveal significant correlation [36]. (Results are detailed in Table 4).

**Table 4:** Results of published studies on the association between six genes and ischemic stroke in Morocco

Study	Cases	Controls	Genes	Mutations	Genotypes	Methods	Odds Ratio 95% (CI)	*P* value
They, TP et al., 2011 [40]	91	182	*MTHFR*	*C677T*	T allele and Stroke	PCR	1.1 (0.59–2.04)	0.303^Ω^
					T allele and atherothrombotic subtype stroke		2.1 (1.17–3.8)	0.012^Ω^
They, TP et al., 2013 [41]	91	182	MTHFR^*^ *F2*	*C677T* *G20210A*	*677TT/CC* *20210GA*/*GG*	PCR-RFLP	4.99 (1.75–14.2) 5.29 (1.63–17.1)	0.001^∏^ 0.005^∑^
Diakite, B et al., 2014 [39]	165	182	*eNOS*	*G894 T*	TT vs. GG + GT Recessive	PCR-RFLP	2,68 (1,08-6,70)	0.034 ^Δ^
					GT + TT vs. GG Dominant		1.78 (1,16–2,73)	0.009 ^Δ^
					T vs. G Additive models		1.71 (1,21–2,43)	0.003 ^Δ^
Diakite, B et al., 2015 [37]	170	211	*FV*	*C2491T*	T allèle	PCR-RFLP	3.77 (2.70–5.25)	< 0.0001^**^
					CT		4.08 (2.55–6.49)	
					TT		8.95 (4.15–19.29)	
Diakite, B et al., 2016 [38]	175	201	*APOA5*	*T1131C*	CC	PCR-RFLP	2.86 (1.24–6.58)	0.014 ^⋿^
					C allele		1.54 (1.01–2.33)	
			*ALOX5AP*	*SG13S114*	TT	PCR-RFLP	2.57 (1.49–4.83)	0.009 ^⋿^
					T allele		1.59 (1.16–2.19)	0.008 ^⋿^
Balar, K et al., 2014 [36]	165^†^		*MTHFR*	Facteurs de risque ^‡^	CT/TT	–	–	NS ^

*PCR-RFLP* Polymerase Chain Reaction-Restriction Fragment Length Polymorphism, ^*^Synergistic effect of MTHFR C677T and F2 G20210A polymorphisms on ischemic stroke. ^†^Prospective study, ^‡^Age/Diabetes/High Blood Pressure/Smoking/Alcoholism/ Cholesterol, NS: Not significant, ^Ω^Hardy– Weinberg equilibrium tests were performed for MTHFR C677T polymorphism separately among cases and controls with the use of Fisher’s exact test, ^∏^Univariate analysis for the combined polymorphisms, ^∑^multivariate analysis for the combined polymorphisms, ^Δ^The law of the genotype distribution for G894 T eNOS polymorphism among disease cases and controls were performed with Hardy–Weinberg equilibrium test with the use of χ2 test, ^**^Significant; HWE, Hardy–Weinberg equilibrium, ^⋿^Hardy–Weinberg equilibrium test with the use of χ2 test or Fisher test, ^the multivariate analysis (logistic regression)

### TOAST etiological classification of ischemic stroke in Moroccan studies

The most prominent etiology is atherosclerosis of large arteries according to (*n* = 16) studies (16–57.8%) [16, 17, 23, 25–28, 30, 32–35, 37–40]. The cardioembolic origin comes second in (*n* = 17) studies (8.8–50%) [16, 17, 20, 23, 25–28, 30, 32–35, 37–40], undetermined causes were present in (*n* = 12) studies (5.5–34%) [16, 17, 23, 25–28, 30, 32, 33, 35, 40], lacunar ischemic stroke was reported in (*n* = 12) studies (0–39%) [16, 17, 23, 26, 28, 30, 32, 35, 37–40] and other identified causes are recorded in (*n* = 13) studies (0–27.4%] [16, 23, 25, 27, 28, 30, 32, 33, 35, 37–40].

Concerning the etiological category “Other identified causes”, five studies have specified the pathologies involved in the ontogeny and the occurrence of ischemic stroke conditions [25, 27, 28, 30, 35]. The first study was performed by Chraa (2010), and has reported 14 cases of syphilitic arteritis, 12 cases related to disorders of clotting factors, 5 cases of arterial dissection, 4 cases of systemic diseases, 4 cases of migraine, 1 case of chemotherapy, and 1 case of human immunodeficiency virus . The second study was conducted by Chtau (2012) and revealed that 2% of all cases were related to arterial dissections. The third was done by Rachdi (2012) and who reported 1 case of Vaquez disease. The fourth study by Saraya (2013) revealed 1 case of polycythemia, 1 case with interhemispheric Meningioma, a toxic IS after Cannabis consumption, and an IS after cerebral angiography as part of the assessment of a C3 Neuroma. The last study was conducted by Rachdi (2015) and showed that 5% of all cases were related to carotid stenosis when it was greater than 50%. However, six other studies did not identify the “Other identified causes” [32, 33, 37–40].

With respect to the young population (15–45 years old), the undetermined causes were identified in four studies. The first study was by Mbagui (2009) with a percentage of 29% [29]. The second by Chraa et al. (2014) with a percentage of 40.6% [22]. The third by Ibouajbane (2014); 55% of all cases were linked to undetermined causes [31]. Finally, Allaoui et al. (2018) reported a percentage of 24% [43].

The IS of cardioembolic origin was also highlighted in four studies; the first study was performed by Mbagui (2009) and reported a percentage of 21.5% [29]. The second by Ibouajbane (2014) showed a percentage of 15% [31]. Interestingly, the third study was by Chraa et al. (2014) revealed that 33.6% of all cases were present with cardioembolic source [22]. By contrast, the fourth study by Allaoui et al. (2018) reported a percentage of 4% [43].

As for the other determinant causes, they were identified in (*n* = 4) studies (Mbagui, 2009; Chraa et al., 2014; Ibouajbane, 2014; Allaoui et al., 2018) with 21.5%, 14.1%, 15% and 72% respectively [22, 29, 31, 43].

Concerning the details on the etiological class “other specific causes”, four studies specified the causes involved in the occurrence of IS in young population. The first by Mbagui (2009) highlighted the implication of blood diseases, vasculitis, oral contraception and carotid dissections with percentages of 35%, 25% (2 cases of Behcet, 1 case of Takayashu, 2 cases of undetermined vasculitis), and 15%, respectively. In addition, 1 case of sneddon syndrome, and 1 post-partum cases were reported [29].

The second by Ibouajbane (2014), angiitis accounted for 5% of cases, hematological disorders for 5% of the cases, 1 case was observed during pregnancy and especially during the sixth month, and thrombophlebitis in another case [31]. The third by Chraa et al. (2014) described 11 cases of syphilis, 3 cases of carotid dissections, 2 cases of coagulation protein deficiency (C), 1 case of sneddon syndrome, and 1 case of anti-phospholipids’ antibody syndrome [22].

The fourth study by Allaoui et al. (2018) described a percentage of determinate causes of 72%. The etiologies in this study were dominated by systemic lupus (32%) associated with antiphospholipid syndrome (80%), Behcet’s disease (16%), Takayasu’s disease (12%) [43].

With respect to atherosclerosis of large arteries, it was reported in (*n* = 3) studies; Chraa et al. (2014), Ibouajbane (2014) and Mbagui (2009) with proportions respectively of 11.7%, 12.5% and 25.8% [22, 29, 31].

Lacunar IS was found in (*n* = 2) studies, including Mbagui (2009) and Ibouajbane (2014) with ratios of 2.1 and 2.5%, respectively [29, 31]. In Allaoui et al. study (2018), TOAST III (lacunary) patients were 73% smokers, 8% had type II diabetes and/or High blood pressure, and 12% had oestroprogestative contraception at the time of diagnosis [43]. (Results are detailed in Table 5).

**Table 5:** TOAST etiological classification of ischemic stroke according to selected studies

Studies	Sample size	Atherosclerosis of large arteries (%)	Cardio-embolic Stroke (%)	Occlusion of small vessels (Lacunar) (%)	Other determinate causes of stroke (%)	Undetermined causes of stroke (%)
Chraa, M., 2010 [30]	352	32,1	28,4	7,38	11,65	20,45
Mbagui., 2009 [29]	93^YIS^	25,81	21,51	2,15	21,51	29,03
Chtaou, N et al., 2016 [23]	52	32	50	0	0	18
Chtaou, N., 2012 [25]	50	44	28	–	2	26
Yonmadji, N., 2016 [32]	1184	57,8	21,4	10	5,25	5,55
Diakite, B et al., 2015 [37]	170	39,4	27,1	6,5	27,1	–
Belkouch et al., 2015 [16]	13	41,3	27	22	0	9,7
Hadi, A et al., 2018 [26]	230	16	32	34	–	18
Diakite, B et al., 2016 [38]	175	39,4	26,9	6,3	27,4	–
Rachdi, L., 2012 [27]	40	39	43,9	–	2,5	14,6
Ibouajbane., 2014 [31]	40^YIS^	12,5	15	2,5	15	55
Chraa, M et al., 2014 [22]	128^YIS^	11,72	33,59	–	14,06	40,62
Diakite, B et al., 2014 [39]	165	56,36	32,12	4,84	6,66	–
Bendriss, L et al., 2012 [17]	110	28	18	39	–	14,5
Chraa, M et al., 2017 [20]	442	–	28,4	–	–	–
Saraya, T., 2013 [35]	242	18,6	25,6	17,8	20,3	17,8
They TP et al., 2011 [40]	91	46,2	8,8	26,4	12,1	6,6
Azdad, O., 2012 [33]	1300	53,8	30,7	–	1	13,8
Rachdi, L et al., 2015 [28]	439	21	24	16	5	34
Rhissassi et al., 2010 [34]	342	52,1	27,6	–	–	–
Allaoui, A et al., 2018 [43]	25^YIS^	–	4	–	72	24

YIS: Ischemic stroke of young adult (15–45 years)

### Prehospital delay in patients with ischemic stroke in Morocco

Since the notion of time is crucial in the management of cerebral ischemia, (*n* = 5) studies evaluated the prehospital delay, which consists of the time extending between the time of the symptoms onset and the patient arrival to the emergency department of the different hospital structures [26, 28, 32–34]. In this perspective, a minimum prehospital average delay was 26 h [Extremes: 15 Minutes- 8 months] according to the study by Azdad (2012) [33] and a maximum mean prehospital delay 61.9 h [Extreme: 0.5 h-216 h] which was listed in the Yonmadji (2016) study [32]. (The results are detailed in Table 6).

**Table 6:** Mean prehospital stroke delay (symptom onset to emergency department arrival) and percent arriving in 3, and after 24 h in Morocco publication

Studies	Study dates	Location	Population reported on	Delay in hours	Per cent arriving in	
					< 3 h	> 24 h
Rhissassi et al., 2010 [34]	During the year 2009	UHC Hassan II Fes	342^IS^	61	–	–
N A-Y AMESSAN., 2010 [15]	November 2009 to April 2010	SH Rabat	46^IS^	–	28	–
Chraa, M., 2010 [30]	January2000 to December2009	UHC Marrakech	352 ^IS^	–	5	–
AZDAD, O., 2012 [33]	01/01/2009 to 01/2/2010	UHC Hassan II Fes	1300 ^IS^	26	9,5	–
Bendriss, L et al., 2012 [17]	January 2005 and August 2008	Cardiology MHAM	110 ^IS^	–	–	41
Saraya, T., 2013 [35]	January2009 to December 2011	MVMTH Rabat	242^IS^	–	4,5	–
RACHDI, L et al., 2015 [28]	June 2014 and December 2014	UHC Hassan II Fes	439 ^IS^	27	–	–
Yonmadji, N., 2016 [32]	January2013 to December 2014	UHC Hassan II Fes	1184 ^IS^	61,9	12,2^a^	68,3
Hadi, A., 2018 [26]	January2010 to December 2014	Cardiology MHAM	230 ^IS^	36	–	–

*IS* Ischemic stroke, *UHC* University Hospital Center, *SH* Specialty Hospital, *MHAM* Military Hospital Avicenna Marrakech, *MVMTH* Mohamed V Military and Training Hospital

^a^Patients received within 4.5 h after onset of symptoms

Concerning the consultation period of young patients, It was assessed in two studies [29, 31]. Mbagui (2009) and Ibouajbane studies (2014) reported 134.4 h and 342 h, respectively [29, 31]. Moreover, Allaoui et al. (2018) showed that the delay between the onset of symptoms and the performance of the first cerebral imaging exceeded 12 h in 100% of cases [43]. No study investigated the factors influencing the consultation and admission time of patients with IS.

### The percentage of patients with thrombolyzed ischemic stroke in Morocco

Four studies conducted at the neurology department of the Hassan II University Hospital of Fez had focused on the thrombolysis management. The proportion of thrombolysed patients ranged from 1.8% in the Azdad study (2012) [33] to 2.9% in the Rachdi (2015) study [28]. In addition, two studies by Yonmadji (2016) and Daouda et al. (2018) revealed two medium proportions of thrombolysed patients of 1.94% and 2.8%, respectively [24, 32].

### Mortality in the acute phase and mortality in the chronic phase (3 months) in Morocco

Six studies reported the mortality rates in the acute phase, ranging from 3% in Yonmadji (2016) study to 13% for Chraa (2010) study. In addition, four studies by Saraya (2013), Rhissassi et al. (2010), Chtaou (2016) and Azdad (2012) revealed intermediate values of 5.8%, 9.9%, 10% and 10.8%, respectively [23, 30, 32–35].

The rate of mortality after 3 months onset of the disease was reported in seven studies. Three studies by Bendriss et al. (2012), Rachdi (2015), and Hadi (2018) reported respectively the mortality rates of 5.4%, 10%, and 8%. Four studies by Daouda et al. (2018), Yonmadji (2016), Chtaou (2016), and Rachdi (2012), respectively reported mortality rates of 4.3%, 21.7%, 29%, and 32.5% in IS treated with thrombolysis [17, 23, 24, 26–28, 32].

The mortality during the acute phase in the young population was indicated in (*n* = 3) studies. The first from Ibouajbane (2014) stated a mortality rate of 0% [31], the second by Mbagui (2009) at 1.1% [29] and the third by Chraa et al. (2014) with a mortality rate of 16.4% [22].

For the mortality after 3 months, no study elucidated this parameter.

## Discussion

The average onset age of IS in Morocco was between 49 ± 15.2 and 67.3 ± 9.9 years old. The studies by Mbagui (2009), Chraa (2010) and Chraa et al. (2014) showed that the onset of IS occurs in 12.3%, 28.9% and 36% respectively of the population under 45 [22, 29, 30]. The early onset of IS in Morocco according to the studies could probably be due to the young age of the Moroccan population and the low percentage of the elderly (over 60 years old), which represents 9.4% according to the results of the last national census of the population of 2014 [17, 30, 44]. The young age could be explained by the high frequency of embolic heart disease, rheumatic heart disease and sexually transmitted infections (syphilis, and acquired immunodeficiency syndrome) in Morocco [22, 33]. In addition, cardioembolic disease is considered as the leading cause of IS in the young population in our country due to the preponderance of rheumatic heart disease [43]. Similarly, it may also be due to the very high level of consanguinity and the significant association with the incidence of health conditions in the Moroccan population [45].

Furthermore, the average onset age of cerebral ischemia in Morocco is still lower with respect to the average age of patients admitted for IS in France in 2014 (74 ± 15 years old) [46] and in comparison to the average age identified in a systematic review in the Arab countries (58.5 and 63 years old) [11]. Besides, the average age of the young population ranged between 28 and 39 years, which is in accordance with the results reported in other North African and the Middle Eastern countries [47–49]. Since Morocco is a country from the Middle East and North Africa region (MENA), the average age reported in Moroccan studies is lower than the one described in non-MENA countries. This confirms the results obtained from the results of Safe Implementation of Treatments in Stroke (SITS)-MENA register (in MENA 55 years versus 73 years in Non-MENA) [50].

Most Moroccan studies have reported male predominance, consistent with the results of a literature review in the Arab world, which indicated that men were most often victims of stroke (between 55.9 and 75%) [11].

These findings are also consistent with the results of a literature review at the level of Eastern Mediterranean countries, which has explored higher prevalence of stroke between men compared to women with a sex ratio up to 3.5 [51]. Moreover, the same findings are in accordance with the results reported in the Middle East between 1980 and 2015 reporting gender differences, and that 75% of studies reported a high sex ratio in patients with stroke [52]. This is also confirmed by a recent observational study based on the results of the SITS-MENA register, which showed a male predominance (72%) in MENA countries compared to non-MENA countries (53.6%) [50]. This result could be due to hormonal factors including estrogen, which seems to have protective effects on both the vascular and cerebral systems [53].

In addition to diabetes, HBP is the main risk factor for IS in Morocco, smoking and heart disease represented the other identified risk factors. In the African context more than half of patients with ischemic stroke had HBP [54]. This joins the results of the literature review of studies conducted in the Middle East between 1980 and 2015, showing that HBP was the most common risk factor, followed by diabetes [52]. These findings are also consistent with data collected in the Eastern Mediterranean countries, where the prevalence of HBP was above 50% in 38 studies, diabetes percentage was higher than 25% in 36 studies, and smoking was higher than 15% in 26 studies [51]. The same results are consistent with those found in a recent observational study showing that hypertension, diabetes and smoking were the main risk factors identified in MENA countries [50].

For atrial fibrillation, most of the studies included in the present review demonstrated percentages exceeding 9%. This rate is higher than the proportion recorded in the SITS-MENA register (8.8%), while atrial fibrillation in non-MENA countries accounted for 19.4% [50].

The elevated frequency of these vascular risk factors in Morocco could be due to the phenomena of urbanization (60.3% of the population), and to changes in lifestyle of the Moroccan population [44], as well as to the westernization of the behavior and the food habits between Moroccans [55]. However, a study conducted in the city of Casablanca showed that fast foods have a high composition of sodium and saturated fatty acids and a small concentration of unsaturated fatty acids, contributing thus to the increased prevalence of cardiovascular diseases and stroke in Morocco [56].

Concerning the risk factors of IS between young people, HBP and smoking remains the most reported factors. This can be justified by the fact that Morocco is considered one of the highest tobacco consuming countries in the Mediterranean area with a consumption rate of 15 billion cigarettes each year, and that 42% of men aged between 30 and 39 years old are smokers [57].

Oral contraception is also a preponderant risk factor between the young subjects. These results can be justified in Morocco by the massive use of hormonal contraceptive methods. For this reason, and according to the performance of the national family planning program established in 2015, the pill represents a percentage of 90% of all contraceptive used methods [58]. Similarly, a meta-analysis of 16 studies conducted in America in 2015 showed that oral contraceptives are associated with an increased relative risk of cerebral infarction of 2.75 [59].

Genetically speaking, studies have suggested that *MTHFR C677T* variant could be a determining event of atherothrombotic IS in Morocco, which is perfectly in line with the results reported in a recent meta-analysis showing that the *MTHFR C677T* mutation increased the risk of IS in adulthood, particularly in atherosclerosis of large arteries [60]. The study by Diakite et al. (2014) suggested a statistically significant association between G894 T polymorphism of eNOS gene and IS, which is consistent with the results of a meta-analysis confirming that G894 T polymorphism of eNOS gene is associated with high risk of IS among Asian populations [61]. Moreover, *T-1131C APOA5* mutation could be considered as a genetic risk factor nondependent on IS between the Moroccan population. The same findings were reported in the Chinese population [62]. For *SG13S114 ALOX5AP*, a significant association was observed in subjects with TT and T alleles in Morocco. The same result was obtained for the Iberian population [63].

Regarding the etiological classification of TOAST, the present work showed that atherosclerosis of the major arteries is the most dominant etiology. This is probably due mainly to the increased prevalence of HBP and diabetes in the Moroccan population. HBP and diabetes are estimated at 29.3% and 10.6%, respectively, in addition to poor compliance with antihypertensive therapy or poor glycemic controls, according to the national survey on common risk factors for non-communicable diseases (NCDs) both in 2017 and 2018. In line with this, the percentage of people with HBP who do not take medication in Morocco is estimated at 71.4% [CI: 69.1–73.7%]. Furthermore, the proportion of people who have never tested their blood glucose levels is 63.2% [CI: 61.8–64.6%] according to the same survey on risk factors common to NCDs [64].

The average prehospital delay for patients with IS in Morocco has ranged between 26 to 61.9 h, which far exceeds the therapeutic window recommended by randomized clinical trials [65].This finding is in accordance with the median admission time (31 h) mentioned in a review of literature on the African continent [66]. What is more, it joins the average consultation time mentioned in a prospective cross-sectional study conducted at the Center of Brazzaville University Hospital in the Republic of Congo, where they reported a period of 28.2 h [67]. A longer time period (16 h) was also recorded in a study conducted at the Sahloul Hospital Center in Sousse, Tunisia [68]. These very long delays listed in our review, were collected mainly in the two university hospitals of Fez and Marrakech and could be explained by the lack of knowledge of the early signs of cerebral infarction. Furthermore, this delay of consultation in the Moroccan population could be linked to the lack of awareness [32, 33]. This finding was confirmed by a study conducted at the Mohamed VI University Hospital Center in Marrakech, in which 59.8% of the interviewees could not name any revealing signs of cerebral ischemia [69]. However, these different findings could probably be due to the elevated illiteracy rate within the Moroccan population (32.2% according to the results of the last census) [44]. This could also be explained by the fact that most of the patients arrived at the emergency rooms using personal or common means of transportation (taxi, personal car), and only a minority used ambulance services (3.5%) [24]. From the same perspective, a recent review demonstrated that low awareness of the signs and symptoms of stroke, the shortage of medical transportation, health care staff and stroke management units, as well as the economic cost of the access to brain imaging facilities and thrombolysis were reported as major obstacles to improve stroke care and stroke management in Africa [66].

Concerning the management of IS patients, all thrombolysis-related studies were conducted at the University Hospital of Fez. Therefore, the small percentage of recruited patients could be due, in major part, to the fact that this technique has been recently implemented in the Hassan II University Hospital of Fez [33]. Interestingly, the Daouda study (2017) showed that 11% of cerebral infarcts did not benefit from thrombolysis because they were admitted after 4.5 h [24]. Moreover, the Rachdi (2015) study also showed that the majority of patients were not thrombolysed because of the long consultation time and other interfering factors such as the lack of patient’s awareness about the clinical signs of IS [28].

Hence, the transportation means (car, taxi, private ambulance), the organizational issues of the emergency medical service (EMS), the difficulties in the recognition of symptoms of IS by the patient, the delays in seeking appropriate emergency care and in obtaining an urgent brain scan and the non-coverage of recombinant tissue plasminogen activator (rt-PA) by the Moroccan National Health System are the main causes involved in the non-access of the population to thrombolysis [23].

Moreover, the performance of the Moroccan neurovascular departments is completely in accordance with the data reported in the literature. In this respect, reperfusion treatments (thrombolysis) were administered only to 1–8% of the admitted patients [70]. A recent meta-analysis showed that only 3% (95% CI 2–4%) of patients were thrombolysed [71].

Indeed, raising the public awareness on a large scale on the neurological signs of the disease are therefore an urgent need, and the organization of pre-hospital medical care from the perspective of reducing admission time and increasing the number of thrombolyzed patients [24].

In addition, the present review revealed that acute mortality ranged from 3% to 13%. These results are lower than the rates reported in other Arab and African countries [72, 73]. These rates are below the estimated mortality rate described (18%) in sub-Saharan Africa during the first week of 2013 [74]. These rates are also lower than the one-month overall lethality rates in Middle Eastern countries, which was ranging from 12 to 32% according to a systematic review between 1980 and 2015 [52]. Similarly, this finding was confirmed by a very recent prospective observational study in the MENA region, showing decreased likelihood of patients death in the MENA with respect to non-MENA countries (6.5 versus 9.6%, *p* <  0.001) [75].

Despite the importance of atherosclerotic ischemic and embolic origins in the context of studies of populations, the present critical review demonstrated low rates of lethality and minor rates of occlusion of small arteries [73]. These findings could be due to the implementation of a strategy of diagnosis and effective etiological treatment of cerebral ischemia in the studies we included in our review. Low mortality rates could also be explained by the lack of ischemic stroke studies evaluating mortality rates in Morocco [71]. Three-month mortality in thrombolyzed patients exceeded 20%, according to most studies, which is higher than the mortality rates recorded in a meta-analysis (about 13.4%) [76].

## Limitations

The present review suffered from several limitations; for example, the studies in question cover only five major cities located in the central parts of the country. We still do not have any data about IS in the north and south of Morocco. Thus, it is difficult to draw any conclusions about the general population. Similarly, most studies were conducted at university hospitals, while most of the Moroccan population has limited access to these facilities. Besides, we should consider the limited access to health care services in rural areas, and the fact that half of stroke patients are not treated in hospitals [77], which exclude a wide range of epidemiological data.

Furthermore, the most of the research on IS was only observation-based studies (Case series and cross-sectional studies), so the authors only tried to describe the patterns of the different variables (risk factors, etiologies…) without using any statistical analysis to reveal potential correlations. Also, the very lacking data originated from the nature of the studies and the implemented local logistics.

Another limitation concerns Moroccan hospital facilities, which mostly receive patients with severe stroke conditions displaying consciousness perturbations and severe hemiplegia [77], decreasing the medical attention toward minor strokes.

## Conclusions

The IS remains a multi-factorial debilitating disorder in Morocco. Overall, available data show a concentration of all studies at university hospitals in metropolitan cities. Also, they suggest that cerebral ischemia is characterized by precocious onset (begins at an early age), male predominance, and etiologies and vascular risk factors observed in patients with IS, which is generally similar to findings in other Arab and African countries. What is more, several genetic markers have been suggested as predisposing factors of cerebral infarction in Morocco. The prehospital delays are very long, compared to the different deadlines listed in developed countries with pathways for the management of IS. Therefore, the expansion of epidemiological studies, particularly in other regions of the country will provide an opportunity to sharpen the incidence and prevalence. Finally, it is high time to further investigate the factors associated with long hospital admission delays in this invalidating pathology to increase the level of brain infarction eligibility for thrombolysis.

## Data Availability

All data used in the publication of this work were obtained from published studies. Also, the data supporting the conclusions in this article are available in the additional files.

## Abbreviations

AF: Atrial fibrillation
EMS: Emergency medical service
HBP: High blood pressure
IS: Ischemic stroke
MENA: Middle-East and North African
NCDs: Noncommunicable diseases
PRISMA: Preferred reporting items for systematic reviews and meta-analyses
rt-PA: Recombinant tissue plasminogen activator
SITS: Safe implementation of treatments in stroke
TOAST: Trial of ORG classification 10,172 in Acute Stroke Treatment
YIS: Young ischemic stroke
